# Strategic human resource management and performance in public hospitals in Ethiopia

**DOI:** 10.3389/fpubh.2022.915317

**Published:** 2022-10-20

**Authors:** Philipos Petros Gile, Joris van de Klundert, Martina Buljac-Samardzic

**Affiliations:** ^1^Erasmus School of Health Policy and Management, Erasmus University Rotterdam, Rotterdam, Netherlands; ^2^Higher Education Institutions' Partnership, Addis Ababa, Ethiopia; ^3^School of Business, Universidad Adolfo Ibáñez, Santiago de Chile, Chile

**Keywords:** Ethiopian public hospitals, strategic human resource management, employee outcomes, performance, hospital reform

## Abstract

**Background:**

Ethiopian public hospitals struggle to meet health care needs of the Ethiopian population, in part because of the persistent human resources crisis. The health reforms and tight human resource management (HRM) regulation of the government have resulted in limited progress toward addressing this crisis. This study aims to analyze how the strategic HRM practices adopted by Ethiopian public hospitals influence employee outcomes, organizational outcomes, and patient outcomes.

**Methods:**

Structured interviews were conducted with 19 CEOs and HR managers from 15 hospitals. Four focus groups were also conducted, with 38 participants (professionals and line managers). The transcripts were thematically analyzed using ATLAS.ti 8. Deductive coding was used based on the Contextual SHRM framework, while remaining open for codes that emerged.

**Results:**

Intended HR practices are influenced by mandatory strict government regulations. Nevertheless, some room for self-selected (bundles of) HR practices is perceived by hospitals. Employees perceive that governmental steered HR practices may not match its intentions due to implementation issues, related to lack of support and skilled management and HR professionals. These problems are leading to dissatisfaction, demotivation, moonlighting and turnover of skilled professionals and perceived to consequently negatively influence performance (i.e., patient satisfaction and waiting time).

**Conclusions:**

There are considerable contextual challenges for SHRM in Ethiopian public hospitals. Hospital management can benefit from having more leeway and from exploiting it more effectively to improve actual and perceived strategic human resource management practices. Adoption of commitment based practices, in addition to mandatory control oriented practices can help to motivate and retain health care professionals and consequently improve outcomes.

## Introduction

The federal state Ethiopia currently comprises eleven regional states and two city administrations (1). Ethiopia ranges 174th out of 188 countries in the Human Development Index 2017 (2) and its health system faces severe challenges, even in comparison to other sub-Saharan countries (3). The health work force lacks the numbers and skills to meet the need of the population of ~120 million (4) and deliver the desired outcomes for patients (5).

Ethiopian public hospitals play a pivotal role in the struggle to deliver the desired patient outcomes, in part because of the persistent human resources crisis. In cognizance of the human resource shortages, the Federal Ministry of Health (FMOH) devised and implemented health reforms by educating and allocating larger numbers of skilled health professionals (3, 6). For instance, the reforms intend to increase minimum staffing standards for various health facilities including hospitals from well below 200.000 to 230.794 by 2020 and 346.649 by 2025. In addition, the country health human resources strategy sets a vision for “well qualified, committed, compassionate, respectful and caring health workers” (6). This lead to an urgent call to develop a Human Resources for Health (HRH) strategy by healthcare organizations that fosters a healthy, committed and respectful workforce, addresses skills gaps, motivation, satisfaction, turnover, and incentives and thereby improve patient outcomes (3).

Unfortunately, the reforms have struggled to deliver improvements on pre-set employee outcomes (such as job satisfaction), hospital performance (such as waiting times), and patient outcomes (such as patient satisfaction) (7). This manuscript analyses these struggles through a strategic human resource management (SHRM) lens, as further motivated and detailed below.

Strategic HRM has received considerable attention in recent decades because of its importance for organizational success (8, 9). It entails a long term, coherent, plan for employees to be hired, managed, motivated, skilled and developed with the intention of achieving the organization's goals (8–11). As no single HRM practice is able to achieve all organizational goals, multiple HRM practices tailored to the specific organization are needed. In practice, there may however be considerable differences between the intended HRM practices, the actually implemented HRM practices, and the HRM practices as perceived by employees (12). The first difference arises when bundles of HRM practices implemented by management don't match the original intentions. Subsequently, the experiences and perceptions of employees may again be different, as evidence by a variety of studies (13–15). Ultimately, these perceived HRM practices determine employee outcomes and organizational outcomes in general (16).

**Figure 1:**
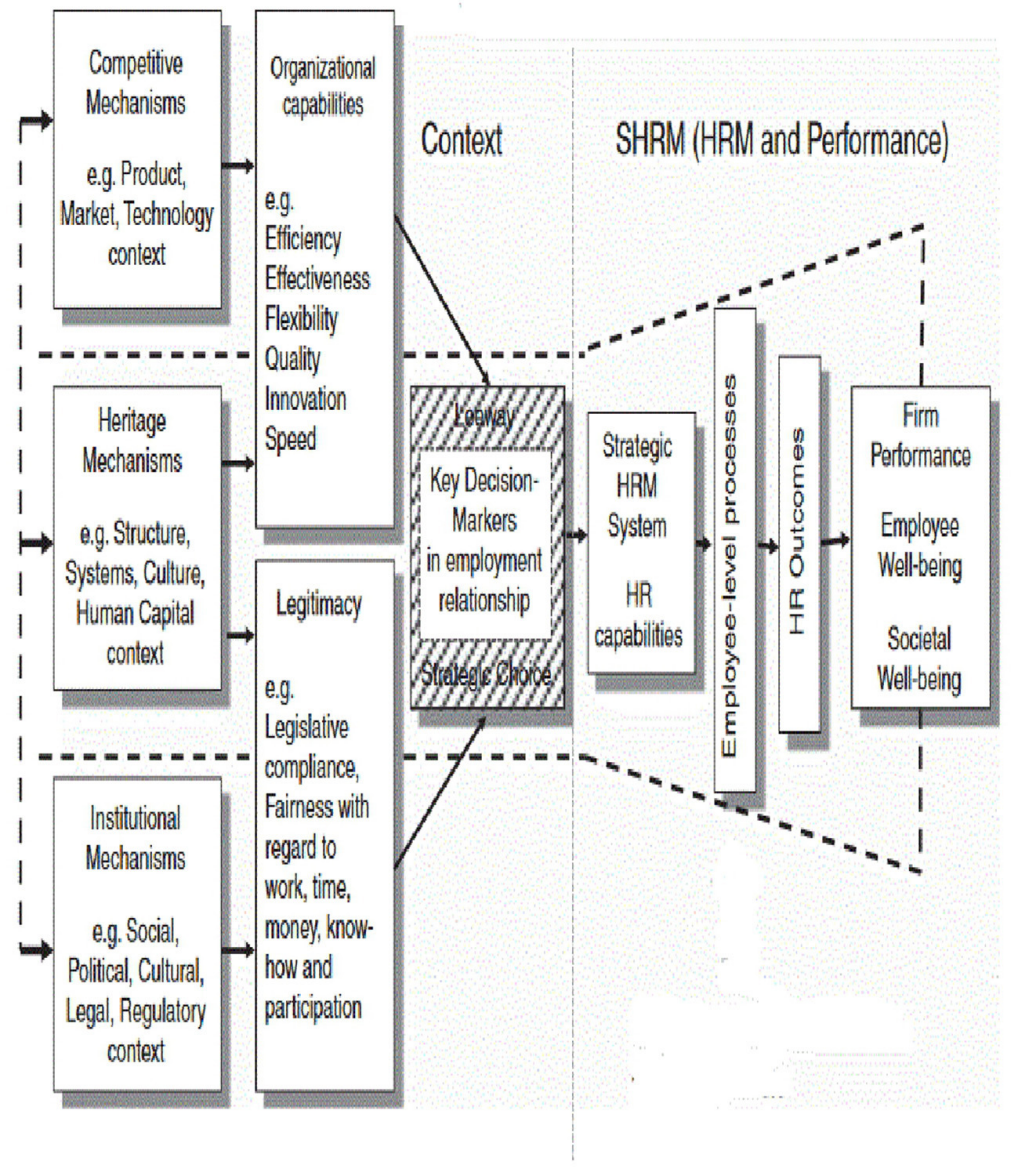
SHRM framework, based on the contextually-based HR theory (14).

The right-hand side of the Contextual SHRM Framework (see Figure 1) provides an additional perspective capturing how SHRM relates to employee outcomes (e.g., commitment, motivation, retention, presence, satisfaction) and (subsequently) to organizational and patient outcomes. Paauwe's SHRM framework synthesizes various other strategic HRM frameworks and has formed the basis for various studies on SHRM [see e.g., (17, 18)]. This study builds on this framework and literature and complements a companion study that focuses on the contextual factors for Ethiopian public hospitals using the (the left-hand side of) the same framework (19).

The framework can serve to explain the difficulties Ethiopian public hospitals experience to improve HRM outcomes, and subsequently organizational outcomes and patient outcomes (14). It guided our study to analyze how the SHRM practices adopted by Ethiopian public hospitals influence employee outcomes, organizational outcomes, and patient outcomes. Our research questions regarding Ethiopian public hospitals therefore are (a) how does SHRM evolve in Ethiopian public hospitals, (b) how do HRM practices influence employee outcomes, and (c) how do the resulting employee outcomes relate to organizational and patient outcomes?

## Materials and methods

A qualitative study design was used to address the explorative research aim and conducted through structured interviews with respondents from public hospitals in Ethiopia. The interview structure was derived from the complete Contextual SHRM framework by Paauwe, which consist of the Context part (left side) and the SHRM part (right side) (see Figure 1). Our research findings on the influence of the contextual factors on strategic HRM have been published separately (20 = 24). The present paper focuses on the SHRM part of the framework, referring to the relationship between HRM and Performance. Both studies have used the same methods.

### Data collection

We selected 15 hospitals by purposive sampling. We aimed to select public hospitals that are representative for the Ethiopian public setting. We thus approached a number of hospitals that differed in hospital level (i.e., general, teaching, specialized, primary), geographical setting (i.e., regional big towns, regional and rural provincial settings, city government of Addis Ababa), and governance (i.e., federal and regional level governments). Table 1 provides details of all the participating hospitals. Data were collected between March and September of 2019.

**Table 1:** Hospital characteristics of participating hospitals.

**#**	**Hospital name**	**Level**	**Geographic location**	**Governing body (Public)**	**Established**	**Bed size**	**Staff size**	**Population served**
1	Dupti Hospital	General	Afar Region	Regional Government/Health Bureau	1958	124	292	1.8 mill
2	Arbaminch Hospital	General	SNNP Region	Regional Government/Health Bureau	1961	238	760	1.5 mill
3	Wolaita Soddo Hospital	General teaching	SNNP Region	Central Government: Federal MOH & MoSHE	1928	347	1297	1.5 mill
4	Alert Hospital	General	Addis Ababa City	Central Gov't: Federal MOH	1934	300	1056	1.5 mill
5	St Peter Hospital	Specialized	Addis Ababa City	Central Gov't: Federal MOH	1953	300	1064	5 mill
6	Ghandi Hospital	Specialized	Addis Ababa City	City Government	1958	144	433	1.8 mill
7	Ras Desta Hospital	General	Addis Ababa City	City Government	1931	200	617	1.5 mill
8	St Paul's Hospital	Specialized teaching	Addis Ababa City	Central Government: Federal MOH & MoSHE	1969	700	3866	1.5 mill
9	Adama hospital	General teaching	(East) Oromia Region	Regional Government/Health Bureau	1946	400	710	1.5 mill
10	Mojo Hospital	Primary	(East) Oromia Region	Regional Government/Health Bureau	2015	80	181	35.7095
11	Ambo Hospital	General	(West) Oromia Region	Regional Government/Health Bureau	1946	88	344	1.5 mill
12	Guder Hospital	Primary	(West) Oromia Region	Regional Government/Health Bureau	2015	50	194	124.500
13	Hawassa Hospital	Comprehensive specialized teaching	SNNP Region	Central Government: Federal MOH & MoSHE	1998	350	2257	1.8 mill
14	Tulla Hospital	Primary	SNNP Region	Regional Government/Health Bureau	2017	80	211	114.200
15	Shashemene Hospital	Specialized	(South) Oromia Region	Regional Government/Health Bureau	1948	306	391	2.9 mill

MoSHE, Ministry of Science and Higher Education; MOH, Ministry of Health; SNNP Region, Southern Nations, Nationalities, and Peoples' Region.

Interviews were conducted with purposively selected respondents who fulfill different positions and are knowledgeable of HRM practices and outcomes.

Our aim was to interview the executive board member responsible for HRM and the head of the HRM department of each hospital. In case these respondents were not available, we tried to interview alternative respondents with the corresponding responsibilities (such as HR directors of health bureaus). We are not aware of any unwillingness to participate among the approached respondents. In total, we were able to interview 19 respondents. Table 2 shows the respondents' characteristics.

**Table 2:** Respondents characteristics interview.

Region	Addis Ababa	6
	Afar Region	2
	Oromia Region	5
	SNNPR Region	6
Hospital type	Specialized	5
	General	11
	Primary	3
Position	CEO	6
	Head department	13
Educational background	Bachelor nursing	1
	Bachelor management	8
	Master health	2
	Master management	6
	Medical doctor	1
	Bachelor law	1
Organizational tenure	<2 years	0
	2–5 years	3
	5–10 years	6
	>10 years	10
Years in current position	< 2 years	9
	2–5 years	8
	5–10 years	1
	>10 years	1
Gender	Female	6
	Male	13
Total respondents		19

After completing the individual interviews, we conducted four Focus Group Discussions (FGDs) with 38 participants in total. These discussions followed the same topic list as the individual interviews and served to triangulate, validate and clarify especially the line management and employee perceptions. The focus groups procedure and FGD guides are presented in Appendix 4 under Supplementary Data for further insights. The participants of the FGDs (details presented in Table 3) were clinical staff, coordinators, and line managers for nurses, physicians and of quality and planning departments.

**Table 3:** Focus Group Discussion participants characteristics.

Region	Addis Ababa	10
	Afar Region	10
	Oromia Region	6
	SNNPR Region	12
Hospital type	Specialized	22
	General	16
	Primary	0
Position	Head/ team leader	18
	Coordinator	15
	Clinical staff	5
Educational background	Bachelor nursing	10
	Bachelor management	14
	Bachelor Law	1
	Medical Doctor	1
	Master management	4
	Master health	8
Organizational tenure	< 2 years	9
	2–5 years	11
	5–10 years	17
	>10 years	1
Work experience	< 2 years	1
	2–5 years	6
	5–10 years	17
	>10 years	14
Gender	Female	9
	Male	29
Total respondents FGD		38

The topic list and structured interview guideline (presented in Appendix 1) was based on document analysis of FMOH Regulatory documents/ policies, Hospital Reform Manuals/Guidelines/Requirements, Health Account and National HRH strategic Plan (6, 20–22) and on Paauwe's framework (14). The interview guideline was piloted in three Ethiopian hospitals by the first and second authors and subsequently revised.

### Data analysis

All interviews and FGDs were audio-taped and transcribed verbatim after ensuring (written) consent from all respondents. The transcripts were analyzed using ATLAS.ti 8 to conduct a thematic analysis (23). The analysis followed the following steps, in which all authors were involved:

***Step 1*** The authors familiarized themselves with the data by (re) reading transcripts and identifying essences and patterns of meaning, issues of potential interests.

***Step 2*** An initial coding scheme was developed to generate topics of interest. These initial codes were identified following a deductive coding approach based on the Contextual SHRM framework.

***Step 3*** We verified whether the initial list of codes covered the key elements of the framework and resolved any gaps.

***Step 4*** Broader code groups were created for related codes. Sub-groups were created for code groups with large number and variety of codes. The researchers remained open for codes that inductively emerged from the data and were not based on the Contextual SHRM framework (e.g., poverty, moonlighting).

***Step 5*** Code groups were combined into agreed broader themes which were based on similarities and (visualized) linkages in data and on the framework.

***Step 6*** The final themes were analyzed and synthesized into results as presented below.

### Ethical approval

The research design and interview protocol received ethical approval (Approval NO. EPHI-IRB-131-2018, Date: 31 Dec 2018) from the Ethiopian Public Health Institute. All interviews and group discussions were recorded following the protocol, after respondents have given their explicit informed consent.

## Results

### The strong governmental impact on SHRM

The Ethiopian government tightly regulates human resource management in Ethiopian public hospitals. This tight regulation for instance takes shape through the mandatory HRM checklist. The extensive government regulatory requirements elaborate the guidelines in hundreds of pages [e.g., (20, 21)^1^] addressing topics such as job descriptions for every position, regular satisfaction surveys, documentation of staff files, and policies and procedures for performance appraisal and feedback, recruitment, promotion and transfer, and for occupational health and safety policies. Moreover, the corresponding hospital policies must be compliant with federal policies thus further strengthening the control of federal government over human resource management.

Governmental regulations were the main institutional mechanism in place. These regulations also emphasized human resources and were perceived to tightly regulate employee numbers, salaries, and employment arrangements at detailed levels. These regulations were perceived to restrict the autonomy of hospitals regarding SHRM. Regulation-induced differences in allowances and external employment arrangements were among the main concerns of respondents.

All respondents mentioned that government regulations and control are very tight and leave little room for organizational specific SHRM other than to comply. For instance, some respondents mentioned having to spend much time and energy on mandatory HRM policies and practices for certain group of professionals as newly introduced by the central government.

*“…the hospital is implementing the newly introduced TID HR strategy for nurses only [with the] working schedule apportioned into three [shifts], each eight working hours within 24 hours range, which is not positively regarded by nurses as such referential / discriminatory treatment limits their right to claim for allowance incentives (FG4).”*

Especially relatively poorly resourced rural hospitals reported difficulties to comply with all mandatory practices.

*“Our hospital is not supported with digital technology for controlling staffpresence/absenteeism, moonlighting and payrolls… so we are using paper system ofattendance control that require all staff sign attendance form twice per day rather than using finger print scanner and not yet implementing technology based pay through electronic banking system (R17).”*

“*Though the hospital reform guideline requires accurate documentation of staff file, ourhospital is yet to implement functional HRIs system for staff, due to less attention to invest on skills based training for existing staff on this new technology from regional government. The hospital is implementing tedious /laborious and inefficient staff file documentation HR practices (R13).”*

Within this context of government prescribed HRM practices, the HR departments reported to experience difficulties because of lack of support by the government, senior management, or both. For instance, some respondents viewed that the HR policy set by the government required skills and authority of HR directors and department heads that were lacking in reality:

*“We are facing conflicting HRM systems in this hospital because HR manager and staff inthe department are not properly trained and lacking HR qualifications. The government knows this as it is repeatedly reported to the ministry but no attention [is] given to find solution for this concern… (R4).”*

*“…[the] actual authority of [the] HR department manager in this hospital is against what is expected from him as per the hospital reform guideline which gives power and responsibility to practice all HR activities in managing workforce issues and support improving staff motivation and wellbeing. This is the most difficult HR practice as the managers—even in many public hospitals where I met and share experiences with HR managers during annual health sector performance review meetings organized by the ministry—lack the power to influence top leadership (R12).”*

Employees and line managers participating in the focus group discussion perceived that the implementation of governmental HR policies may not match the intentions.

“*Unpleasant perceptions… toward actual HR practices that are unfairly implemented including allowances, salary increment …. Which dissatisfies them and [is] contributing to poor engagement, commitment or dedication to perform (FG3).”*

In addition, governmental decisions are perceived to prohibit the implementation of the intended strategic HRM plans agreed between government and the hospital in annual plans. For instance, respondents mentioned

*“After scrutinizing annual staffing forecasts for all departments and approval from ourhospital management, my hospital only hires 30% of the corporate HR Plan submitted tothe ministry of health. This is a major cause for shortages in skills and staff and ultimately hampering well being and hospital performance (R4).”*

*“Organizational level HR planning/ forecasting [submitted to the Ministry] from alldepartments is just political motive of making final decision by central government on allallocations of workforce, on every salary increase. With a rationale of managing scarcityor budgetary shortage, the hospital never accomplishes its HR planning… (R1).”*

### Hospital initiated HRM practices

While the HRM activities of hospitals were clearly dominated by following governmental regulations, they also implemented additional self-selected (bundles of) practices. We now first describe some commonly implemented practices and then some uniquely implemented practices.

Several hospitals have for instance implemented motivation-enhancing bundles in which financial and non-financial HR practices are combined. These included ability-and opportunity enhancing HR practices, such as provisioning of on-the-job skills training and facilitating promotion opportunities for well performing staff. Some respondents perceive that employee satisfaction may have benefited from implementing such bundles:

“*…as a result of implementing HR bundles of practices, the overall staff satisfaction inour hospital improved from 75 to 90% in 2018 and 2019, respectively, the positive change is over and above the policy intention (R15).”*

Other respondents mention the importance of bundling financial HRM practices with leadership/management support for motivation and satisfaction.

“*Even if financial issues like wages and allowances are very critical, providing financialincentives alone may not satisfy workforce. Combining financial and non-financialstimuli like leadership support are key to address workload and life condition inducedstresses, burnout and psychological contract to improve employee satisfaction, performance and contribute to organizational outcomes (R10).”*

Among the uniquely implemented HRM practices are free transportation to and from work, free medical care, a supermarket for staff on the hospital premises with fair prices, and a café for staff. Additionally, there were purely non-financial practices such as dedicated staff recognition days, provisioning of free training programs, and certificates of appreciation (see Appendix 3). One of the remote rural hospitals reported to operate a flexible working arrangement schedule for staff to accommodate the harsh local climate, working in the early morning and in the late afternoon.

### HRM outcomes

The majority of the studied hospitals mentioned job satisfaction and organizational commitment as important HRM outcomes. Job satisfaction rates ranged from 28 to 90% over the studied hospitals (see Table 4), many of which appeared to struggle with poor motivation and low satisfaction among the workforce. In addition, respondents reported low commitment, unhappiness, absenteeism, and to be looking for second jobs in the private sector to supplement the low salary (moonlighting) and high turnover.

**Table 4:** Hospital performance measures from HMIS (Sept 2019).

**#**	**Hospital name**	**Job satisfaction (%)**	**Patient satisfaction (%)**	**Waiting time (in minutes)**
1	Dupti Hospital	82	83.6	Nd
2	Arbaminch Hospital	83	84	50
3	Wolaita Soddo Hospital	59.3	79	77
4	Alert Hospital	67	79.8	80
5	St Peter Hospital	58	83.8	86.6
6	Ghandi Hospital	74.1	Nd	46
7	Ras Desta Hospital	nd	Nd	Nd
8	St Paul's Hospital	30	60.6	105.4
9	Adama hospital	60	76	79
10	Mojo Hospital	70	Nd	70
11	Ambo Hospital	64	76	22.6
12	Guder Hospital	90	84	Nd
13	Hawassa Hospital	49.4	78.6	66.7
14	Tulla Hospital	78	76	Nd
15	Shashemene Hospital	27.9	52	20.9

Waiting time is measured in minutes as the total time that a patient spends in a hospital from arrival at the registration desk until the time she/he leaves the facility or last service. Source: Data was collected from HMIS Health Management Information System/data base Units of the studied hospitals, 2019).

In addition to the financial and regulatory reasons mentioned above, many respondents attributed the poor HRM outcomes to the poor working conditions and organizational climate. More specifically, the government and senior management were commonly perceived to pay insufficient attention to job dissatisfaction and commitment issues. Some respondents attribute this to “*…lack of adequate awareness and commitment toward the importance and impact on the behavior and performance of workforce (R16).”*

Respondents share the belief that other employee outcomes such as strong intentions to leave, frequent absenteeism and moonlighting are also driven by poor HRM practices in general and particularly mention the lack of fairness and autonomy.

“*Lack of effective HR strategy/practice to motivate staff and retain them in their workcausing moonlighting and intention for turnover that ultimately [negatively) affectingorganizational capability of efficiency and effectiveness in service provision and healthoutcomes (FG2).”*

The views expressed in this statement also position employee outcomes as a determinant of hospital performance and patient outcomes. Other respondents further lengthened this causal chain backwards to HRM practices:

“*… ineffective HRM practice of giving less attention to HR issues, and leadership incompetence* (R7), … *caused low HRM outcomes [e.g., dissatisfaction, demotivation] and organizational productivity in service delivery and health outcomes including patient satisfaction and waiting time (R14)*.”

**Figure 2:**
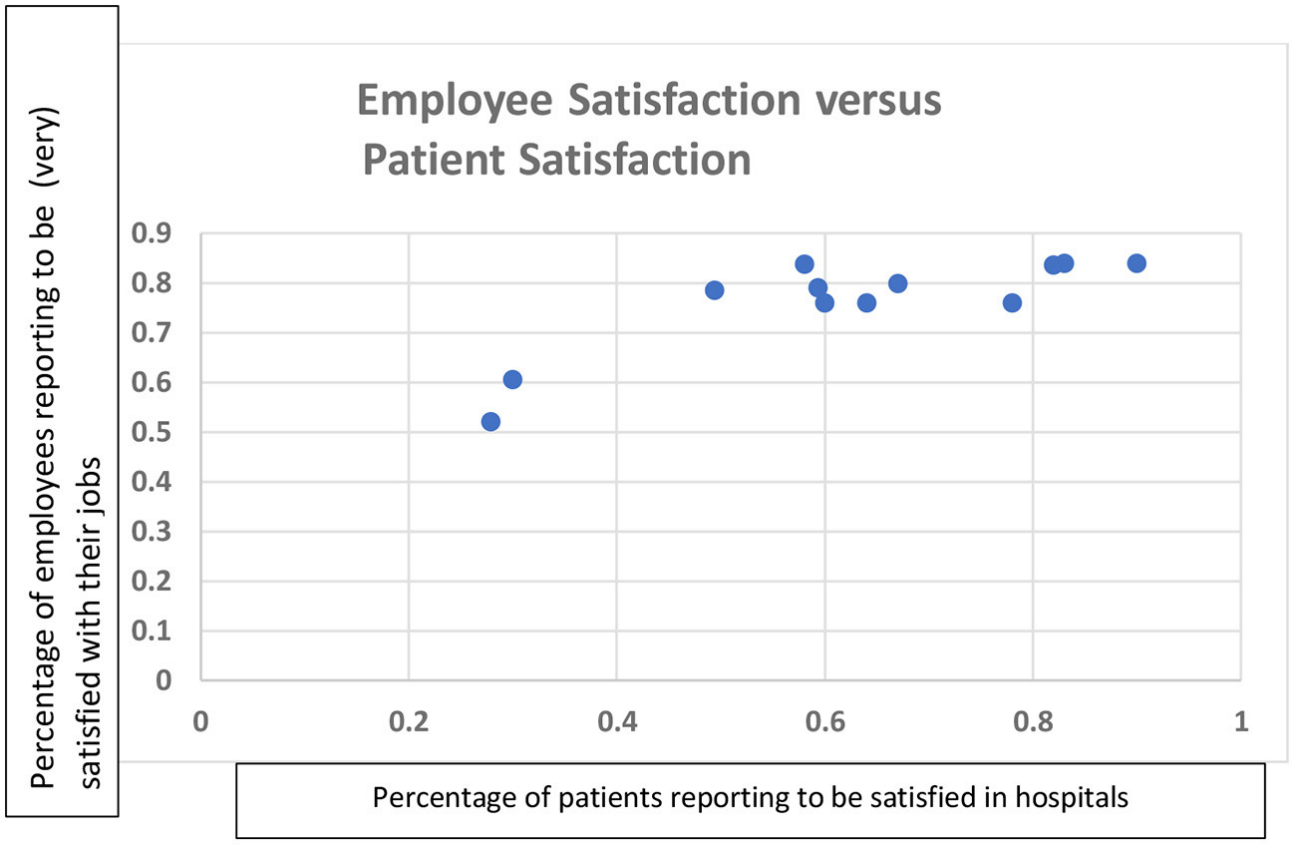
Employee satisfaction vs. patient satisfaction.

**Figure 3:**
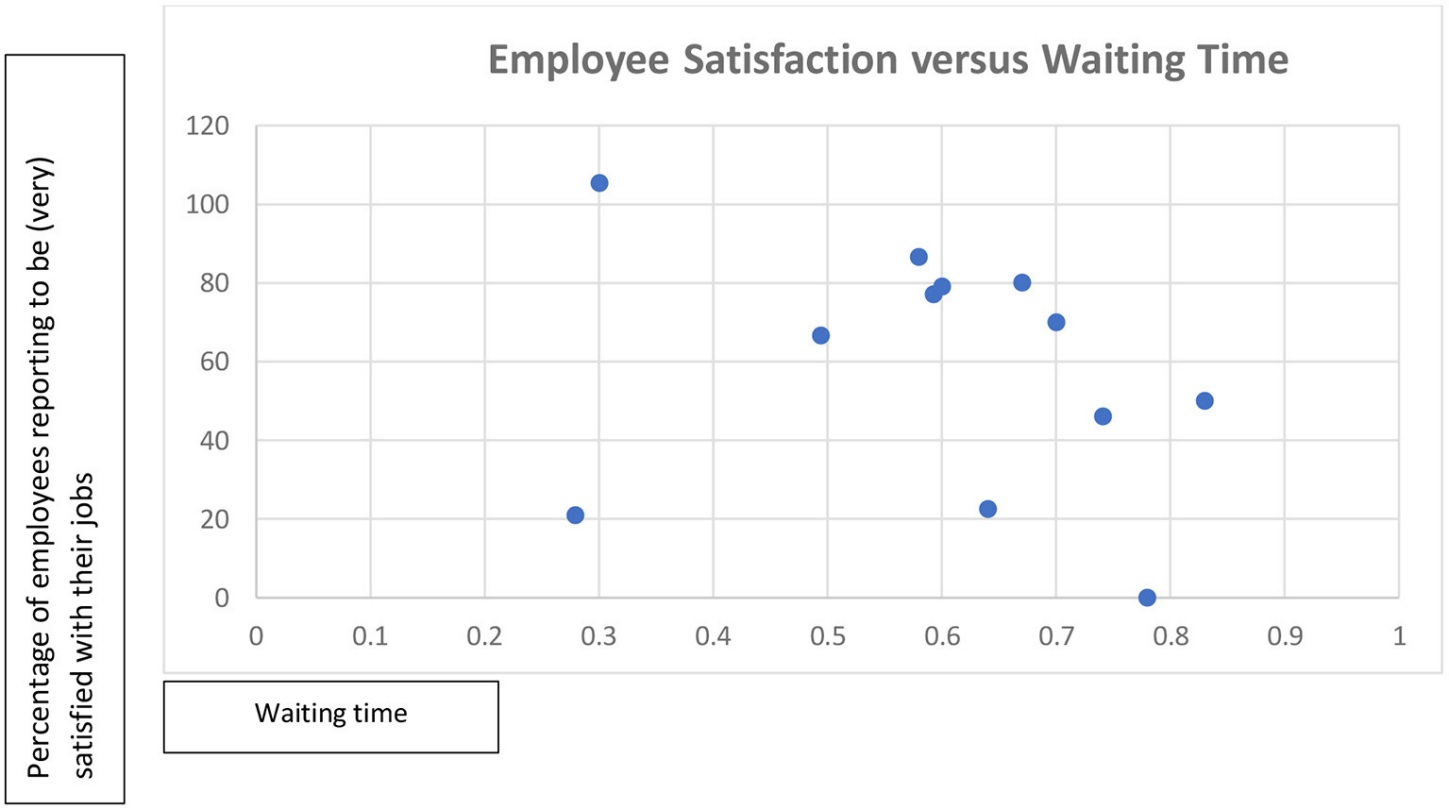
Employee satisfaction vs. waiting time.

Table 4 and the corresponding Figures 2, 3 provide a brief descriptive analysis of data which is collected mandatorily and obtained from respondents' hospital information management systems. Together, they provide some quantitative support for the proposition that higher employee satisfaction is associated with shorter patient waiting time and higher patient satisfaction. However, this is not a conclusive link as patient satisfaction is mostly quite high, even when employee satisfaction is low.

Patient satisfaction was frequently considered as a patient outcome and is typically reported annually. It has been around 80 percent for ten out of the twelve hospitals reporting patient satisfaction scores, whereas the other two score between 50 and 60 percent. Patient satisfaction was also linked to employee satisfaction and HRM practices by respondents. It was positively associated with “leadership competence and workforce commitment” (R1) and with “training” and “employee outcomes” (R7). Regarding the link between HR systems in the studied hospitals and outcomes, some respondents framed this negatively by stating that the poor work conditions, insufficient financial incentives and compensation negatively impact employee outcomes and subsequently patient outcomes, as would be supported by Figure 2.

“*Employee well-being is threatened due to poor attention by policy makers given forHRM. Even if workforce always requesting for well being yet government HR strategy and policy lacks these, the end result has a negative impact on patient outcomes and hospital performance (R12)*.”

Other respondents are of the opinion that patient satisfaction is held up by staff despite HRM practices: “*Surprisingly employees are committed to the patients regardless of the poor conditions with low wages, discriminatory allowances (FG3).”*

Lastly, some respondents link the hospital and patient outcomes back to the regulations and perceive the regulations to fail the goals they are intended to achieve:

“*The hospital is unable to effectively and efficiently deliver quality services due to factors like health regulation induced negative influences on workforce motivation (FG3)…. Policy and regulatory framework on HRM and political force induced budget shortage (R2).”*

The root causes for the challenges to improve patient outcomes are even sought outside of the context of governmental heath policy, i.e., in the broader societal context: “*poor economic development/ poverty situation (are) hindering hospitals to provide standard healthcare and patient outcomes (R3).”*

## Discussion/conclusions

Guided by the Contextual SHRM framework of Paauwe (14, 24) our study sheds light on how HRM system generate employee outcomes and influence organizational and patient outcomes in Ethiopian public hospitals.

Pertaining to the strong government impact on SHRM [see also (20)], our findings provide evidence on how the government tightly regulates HRM in the public hospitals. The government uses the tight regulation and enforcement as instruments to shape the intended HRM that leave hospitals little leeway to intend otherwise and build tailored and distinguishing HRM systems.

Nevertheless, we find that actual HRM practices implemented by managers may deviate from the intended HRM practices because the government has difficulties to provide the financial resources, skilled health professionals, and workforce as specified in agreed annual budgets. Some hospitals are more successful than others in controlling the threatening gaps between intentions and implementation, e.g., by diminishing the shortages, possibly through their political connections.

Differences between intended and actual HR practices have been previously reported in other settings (12, 15). In previous work, the differences often originated at lower management echelons, e.g., through line managers interpretation and implementation of intended HR practices (25, 26). In our study, by contrast, we find that the differences arise at higher management levels as described above. Lower management level mostly restricted themselves to compliant implementation and appeared to deviate only in case hospitals failed to fill their positions with qualified staff. Some studies [e.g., (27, 28)] suggest that intended, implemented, and perceived HR practices must be aligned in order to lead to the desired outcomes. Communication by management is important to this purpose, as it plays a moderating role in the relationship between intended and perceived HR practices.

Only a few Ethiopian hospitals managed to implement HRM practices beyond the struggle with mandatory HRM practices. In these exceptional cases, hospital leadership appreciated and utilized the little existing leeway to append intended HR practices locally and implement actual HR practices that addressed the needs of the workforce. Such practices could take the form of providing subsidized supermarkets for staff, transportation to reduce the cost of commuting, or free health services. They also included skills and opportunity enhancing practices, such as free training and nomination for promotion (29–31). Overall however, our findings suggest that the HR systems of Ethiopian public hospitals tend to extend the control orientation of the government regulations (6, 20, 21) and hardly adopt commitment-based practices, as they are more commonly encountered in Western public hospitals (32, 33). The few successful examples suggest that there is more leeway for organizational level HR than many Ethiopian public hospitals utilize.

The Ethiopian public hospitals which leveraged their leeway mostly did so by providing non-financial arrangement to attract and retain employees, such as flexible working arrangements, training programs and forms of recognition of achievement. These findings from resource constraint public hospitals in Ethiopia confirm previous research from other contexts showing that innovative HR practices often focus on non-financial HR practices [e.g., (34–38)]. The limited uptake may be explained by the perception that financial arrangements are the main priority because of the financial nature of the most important needs of the workforce.

Regarding the perceived HRM practices, our findings indicate that the government motivations to allocate scarce human and financial resources over the health system serving a population of 120 million are recognized and viewed as legitimate. However, the resulting regulations and intended HR practices are partially perceived as unfair and demotivating. These perceptions apply even stronger to the actually implemented HR practices in cases where the implementation of governmental HR policies is viewed to deviate unfairly and in a demotivating manner from the intended HRM of the regulations. This holds particularly true for the financial arrangements, such as salary and other benefits, and opportunities provided for moonlighting. Such negative perceptions of HR practices are exacerbated by perceived incompetence of hospital management and HRM department and inequitable decisions made at hospital level. Thus, the perceived HRM leads to poor employee outcomes such as poor job satisfaction, absenteeism, and high turnover. These poor employee outcomes in turn are perceived to negatively impact other outcomes envisioned to be obtained by the intended HR practices as enforced by the tight government regulations. These mechanisms appear to apply regardless of hospital size, level, location or governance. The thus found differences between perceived HRM outcomes on the one hand and intended HRM outcomes on the other hand have been previously reported [e.g., (12, 16, 26)].

As the perceived HRM practices appear to cause demotivation, absenteeism, and turnover, one might expect these perceptions to subsequently diminish outcomes important for patients such as waiting times and patient satisfaction (36–39). While our findings suggest that employee satisfaction is correlated with less waiting times, we have not found similar perceptions from all respondents on the relations between employee satisfaction to patient satisfaction. Perhaps this is due to mechanisms neutralizing the negative impacts of demotivation, absenteeism and turnover on patient outcomes. Indeed, we have found that patient outcomes provide motivation and purpose and promote employee outcomes, in a direction opposite to the logic of the chain of causality of the SHRM model and the government regulations. These findings support previous research suggesting that the intrinsic motivation of health professionals to serve patients and their professional logic effectively counters external motivators such as the regulations and financial incentives of the management logic (39, 40). An alternative explanation is that patient satisfaction is determined by the perceived service delivery in relation to their expectations toward quality of healthcare (41–43). The legacy of poor performance will then have resulted in low expectations which are easy to meet (42–44), regardless of employee satisfaction.

Based on our analysis, the following policy recommendations may serve the government and hospitals to address HRM challenges found to negatively influence employee and patient outcomes.

1. The government can provide a supporting context for hospitals by providing sufficient funding and ensuring capable hospital managers and sufficient HR personnel to implement intended HR practices correctly and fairly. However, for implemented HR practices to lead to the desired outcomes, it is important that they are perceived in the same way as intended. As management communication plays a moderating role in the relationship between intended and implemented, but also between implemented and perceived HR practices, the government support could therefore include corresponding guidelines on selection and training of managers.

2. By loosening the current tight and centralized government regulations, the government would increase leeway for hospitals to adopt commitment-based approaches and address the needs of their workforce. This would imply enhanced autonomy for hospitals to manage and ensure how the HRM strategy addresses the needs of workforce. This requires a new agency between government and hospitals.

3. Hospital senior management can identify and utilize more leeway for SHRM and adapt internally aligned ability-, motivation-, and opportunity- enhancing HR bundles in their organization.

4. Senior management can in turn empower HR management and line management, make and grant time to clarify HR practices and their importance.

5. Engagement and involvement of professionals in SHRM may serve to close gaps between the management logic of tight control oriented HRM system/strategy and professional logics (to provide quality services) early on: in the intended HR designs. In addition, perceived HR practices can also be shaped by co-workers (next to managers), which requires facilitating interaction among employees.

## Strength and limitations of the study

This study includes a large and varied sample of Ethiopian hospitals covering various geographic locations, rural and urban settings, and central and regional governments. The study engaged various respondents, ranging from experienced administrators and HR managers to team leaders and professionals. The triangulation was further reinforced by using combination of study design entailing interviews, focus groups, and document analysis.

A first limitation of our study is caused by the regional conflicts within Ethiopia that formed delays and restricted travels to the regions Oromia and SNNPR. Although we did collect data in these regions, some factors and mechanisms may have been emphasized less and others more due to the regional conflicts during the data collection. Likewise, the situations and dynamics in Ethiopia have impacted the actual data collection process and lead the researchers to comply with the approved research protocol, rather than consider collection of additional data after verifying data saturation. Hence, we may have missed some factors and aspects of mechanisms, and our study may not be generalizable to all hospitals in the dynamic setting of Ethiopia. Secondly, employee views were not collected through personal interviews but through FGDs. Therefore, the views and perceptions of people on the work floor may be articulated less explicitly in the findings. Future studies may more explicitly explore the employee perceptions of HR practices and outcomes. Thirdly, the study exclusively focused on public hospitals in Ethiopia. Therefore, the generalizability to the private hospitals and other healthcare settings in Ethiopia and beyond may be limited.

## Data availability statement

The datasets presented in this study can be found in online repositories. The names of the repository/repositories and accession number(s) can be found in the article/Supplementary material.

## Ethics statement

The studies involving human participants were reviewed and approved by the Ethiopian Public Health Institute (Approval No. EPHI-IRB-131-2018, Date: 31 Dec 2018). The patients/participants provided their written informed consent to participate in this study.

## Author contributions

All authors listed have made a substantial, direct, and intellectual contribution to the work and approved it for publication.

## Conflict of interest

The authors declare that the research was conducted in the absence of any commercial or financial relationships that could be construed as a potential conflict of interest.

## Publisher's note

All claims expressed in this article are solely those of the authors and do not necessarily represent those of their affiliated organizations, or those of the publisher, the editors and the reviewers. Any product that may be evaluated in this article, or claim that may be made by its manufacturer, is not guaranteed or endorsed by the publisher.

## Footnotes

1 http://www.forsslund.org/StandardHealthFaclitiy/Specialized%20Hospita.pdf (accessed October 22, 2018).
