# Supplementary material for: Strategic human resource management and performance in public hospitals in Ethiopia
Source: Front Public Health. 2022 Oct 20;10:915317. doi: 10.3389/fpubh.2022.915317 (PMC9632433; doi:10.3389/fpubh.2022.915317)
Supplement: Supplementary file 1 [file Table_1.docx]

**Supplementary materials (Appendix ,1,2,3,4)**

**Appendix 1 (Supplementary data) : Topic list and interview guide to study HRM in Ethiopian Hospitals**

Dear Sir/Madam;

To introduce myself and aim of the study, I am …… conducting research on HRM practices in Ethiopian hospitals. The aim of the study is to hear your experiences and opinion so as to advance our understanding on how HRM strategies evolve in the Ethiopian hospitals setting. To this end, I would like to interview you, and thank you in advance for your cooperation in sparing your busy schedule.

I would like to ask you questions about several topics that relate how HR strategy is developed in this hospital. There are no good or wrong answers to the questions. This interview will approximately take 75 minutes of your time. We are asking your permission to audiotape the interview (verbal consent), which do not aim to identify and describe your name, personality but only for research purpose. Indeed, your responses will be kept strictly confidential.

1. **Background and introduction questions:**
   1. Could you tell me something about your job and responsibilities within this hospital?
   2. What is your educational background and experiences in this and similar organizations?
   3. How long do you work in this hospital, in the current position?
   4. What is your relationship to the HR strategy? (possible answers: responsible to develop it, responsible to implement it, ...)
2. **Institutional Mechanisms[from health system, regulation & legal, political, cultural & societal contexts)**

2.1. Which characteristics and developments in the health system impact the way you manage the hospital and especially your employees (professionals and staff)? Think for example of hiring, salary, working hours and conditions,…..

2.2. Which governmental regulations and laws impact the way you manage the hospital and its workforce? (e.g. national labor laws and regulations, regulation for healthcare institutions)

2.3. What political aspects are influential on your management of the hospital and especially of your employees? Could be your direct relationships, or the relationships of your institution, e.g. with Health Bureau, University, Ministry, Politicians,…

2.4.How do societal and cultural characteristics and developments impact your management of the hospital and especially of the employees ? Think for example on (changing) demand and expectations, economic developments, poverty, health literacy,**…**

1. **Heritage Mechanisms related questions:**

3.1. Can you sketch the organizational structure? Is there a dedicated HR department? Where is it positioned?

3.2. What responsibilities are with the HR department? What HR responsibilities are with executive management? What with the line management?

3.3. What is the history of this hospital, especially the recent history? What is special? What are the strengths, especially in workforce, skills, knowledge. What are challenges in HRM, workforce, skills, knowledge,….

3.4. How would you describe the organizational culture?

1. **Questions related to Competitive Mechanisms :**
   1. What services do you offer? What do you offer especially? What is it that you don’t offer?
   2. Can you describe the market, i.e. your customers and your competitors?
   3. What are the main developments and innovations in the market? Which technologies matter?
   4. Which skills are important? Are you competing for skilled personal? Do they leave for the competitor?
2. **Questions related to ‘Organizational capabilities’ and ‘Legitimacy’ related factors :**
   1. Do you achieve the desired health outcomes for your patients (are you effective)?
   2. Are you responsive? Can you instantly address urgent patients? Do you have short waiting times?
   3. Are you efficient? Do you have low cost? OR are you rather focused on being high quality for instance?

5.4. Do you learn new technology and procedures easily? Can you innovate?

5.5. Do you manage to be compliant with all regulations on hospital care? Where are the challenges? How is it related to human resource management?

5.6. Do you manage to be compliant with all regulations for labor / employee relations? Where are the challenges?

5.7. Can you get all work done within the skills and time of the corresponding employees?

5.8. Can you divide work and manage employees equally and fairly?

5.9.Are employees involved in decision making? Do you think they feel engaged? Involved?

1. **Questions related to Leeway or key decision makers in employment relationship**
   1. Who are the key decision makers in general hospital management?
   2. Who decides on HRM (strategy)?
   3. Could you tell me about your mandate/authority?
   4. Who decides on job description? Who on salary system and salary scale for health professionals? On salaries, promotions, allowances, incentives? Moonlighting? Hiring/firing?

6.5. Is HRM important for your hospital and why?

6.6. What are the roles/functions of CEO or General Manager of this hospital?

6.7. If you would like to change the present HR strategy, would this be possible? To what extent? Who would be able to alter HRM? Where can you advance? Where are you bounded?

6.8. What promotes, what hinders the responsibilities of decision maker/s in this hospital?

6.9.Which factors influence the choices and implementation of HR strategy?

**Could you provide additional documents** about this hospital [establishment, organogram, bed size, staff size, HRM policy/strategy/guideline, Hospital Management Committee/Board profile, annual patient load, job- and patient- satisfaction, recognition award (if any), other KPIs reports, …]?

**Abbreviation:** MOH: Ministry of Health; FMHACA**:** Food, Medicine and Healthcare Administration and Control Authority; CRC: Compassionate, Respectful and Caring; KPI: Key Performance Indicators

**Thanks a lot for your time and cooperation**!

**Participant Information Sheet (PIS) and Consent Form(CF)**

***Dear madam/sir,***

Below, I wish to briefly introduce you why this study is being done and what it will involve.

To introduce myself and aim of the study, I am …. conducting research on HRM practices in Ethiopian hospitals, fully funded by Erasmus University Rotterdam and expected to be completed in April 2019. The aim of the study is to hear your experiences and opinion so as to advance our understanding on how HRM strategies evolve in the Ethiopian hospitals setting. To this end, I and the research team member would like to conduct group discussion, and thank you in advance for your cooperation in sparing your busy schedule. I would like to ask you questions/professional experiences about several topics that relate how HR strategy is developed in your and/or other hospital. There are no good or wrong reflections/answers to the questions. This discussion will approximately take 90 minutes of your time. We are asking your permission to audiotape the group discussion, which do not aim to identify and describe your name, personality but only for research purpose. Tapes will be identified only by a code, and will not be used or made available for any purposes other than the research project. These tapes will be destroyed at the end of the study. *Y*our active participation is instrumental and your responses will be kept strictly confidential.

You have been chosen and approached because of your experiences and roles associated to this research and societal implications to hospitals and health system. Taking part is entirely voluntary, beneficial to improve societal health, HRM in hospitals and organizational performance. There is no intended personal benefit (but social) in taking part, has no risks and that refusal or withdrawal will involve no penalty or loss, now or in the future but your information is vital. Thus, I kindly expect you will give genuine information, experiences, ideas or opinions. We would be happy to share a copy of (if published) results where you will not be identified in any report or publication. The project has received ethical approval certificate from the Ethiopian Public Health Institute (EPHI) of the Federal Ministry of Health*.*

**Consent Form**

- I have been debriefed, read and understood the Participant Information Sheet;
- I have been given the opportunity to ask questions and have had them answered to my satisfaction;
- I agree to take part in this research project;
- I understand that my participation is voluntary and I have been informed that I am free to withdraw at any time without giving a reason;
- I understand and agree to consent for EUR and scientific procedures for handling any personal data (e.g. confidentiality, anonymisation, etc.);
- I understand and agree to consent to proposals for data storage, archiving, sharing and re-use for future research;
- I understand and agree to consent to any planned audio or visual recording.

I kindly ask you to sign, print your name and date this form

| **Print your name** | **Signature** | **Date (GC)** |
| --- | --- | --- |
|  |  |  |

**Appendix 2. Human Resources Management Implementation Checklist with scope of practices**

| Standard HRM practices required to be implemented in hospitals | Yes | No |
| --- | --- | --- |
| Policies and procedures for staff recruitment, promotion and transfer have been developed and implemented |  |  |
| Policies and procedures for performance evaluation have been developed and implemented. |  |  |
| Policies and procedures for employee recognition have been developed and implemented | . |  |
| Policies and procedures for training and development have been developed and implemented. |  |  |
| Policies and procedures for compensation and benefits have been developed and implemented. |  |  |
| Policies and procedures for occupational health and safety services have been developed and implemented. |  |  |
| Policies that define when and what type of identification badges and uniforms are worn by staff have been developed and implemented. |  |  |
| A Human Resource plan has been developed |  |  |
| Training Need Assessment has been conducted and Training and Development Plan developed |  |  |
| Adequate budget is allocated for Human Resource Management/Development |  |  |
| The Human Resource head is represented on the Senior Management Team. |  |  |
| Adequate HR professionals are assigned to provide human resources management services |  |  |
| Job descriptions have been developed for each position at the hospital |  |  |
| Staff job satisfaction survey is conducted regularly. |  |  |
| HR Audit has been conducted |  |  |
| Each employee has a personnel file that is maintained by the Human resource directorate/department. |  |  |
| A Functional Human Resource Information Management System (HRIS) has been established |  |  |

Source: Document Analysis of the Ethiopian Hospital Services Transformation **Guideline**, Chapter 17: HRM Section. FMOH. 2016.; EHRIG[ Ethiopian Hospital Reform Implementation Guideline, Federal Ministry of Health, 2010

**Appendix 3**. Differentiated HR practice: combining non-financial and financial-motivation enhancing HR activities (Dupti General Hospital, Afar ) . The pictures documented by the researcher after getting consent from participants and hospital leadership, showing partial view of celebrating annual Staff Recognition Day with financial and non-financial motivation enhancing practices with leadership support


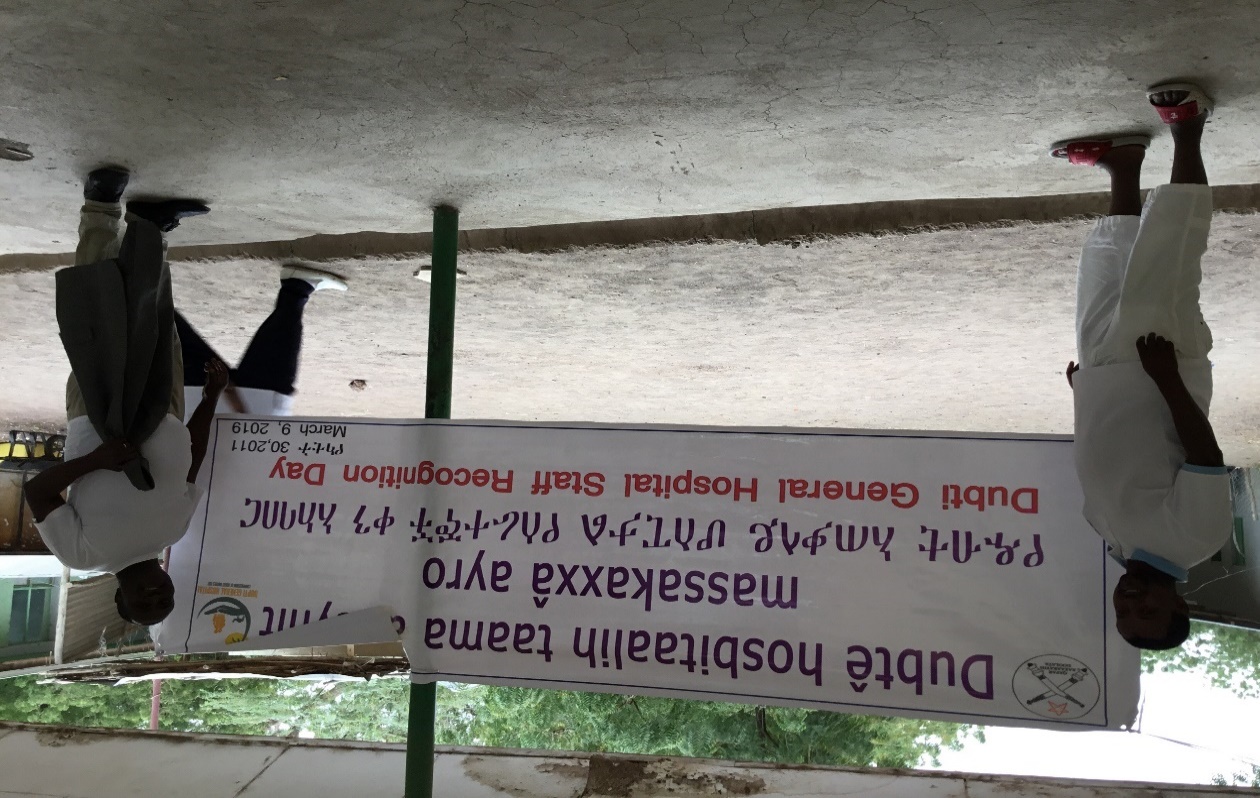


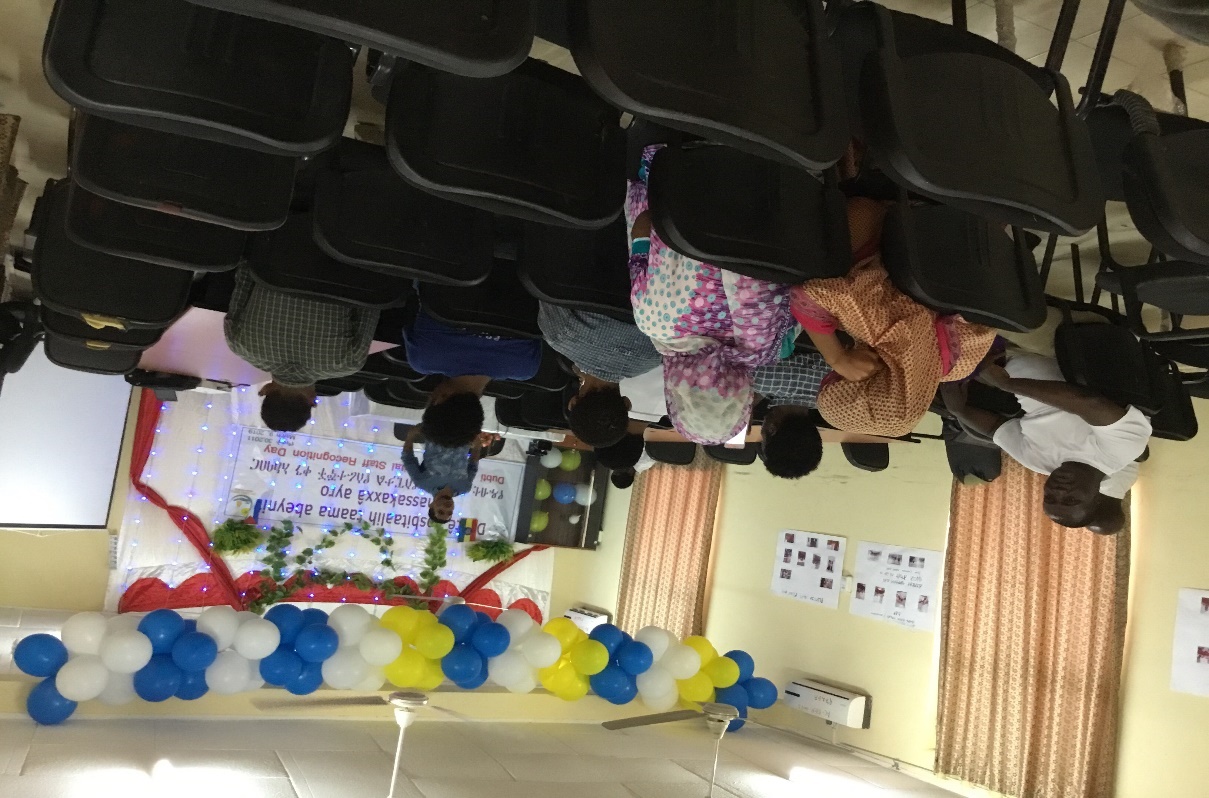


**Appendix 4. Focus Group Discussion (FGD) guide and procedure**

Dear Sir/Madam;

**Introduction and setting the scene :**

Good morning/afternoon and welcome to our session. Thanks for taking the time to join us to talk

about HRM practices in hospitals . To introduce myself and aim of the study, my name is Philipos Petros (introduce research assistant(if applicable), conducting research on SHRM practices in Ethiopian public hospitals. The aim of the study is to hear your experiences and opinion so as to advance our understanding on how HRM strategies evolve in the Ethiopian hospitals setting. I would like to conduct focus group discussion *for validating the interview data analysis*. This FGD will last about one hour.

You've probably noticed the microphone/voice recorder. Following scientific standard, this discussion will be tape recorded; after you have given your explicit consent (a page information sheet and consent form is annexed for your signature after reading). There are no right or wrong answers, only differing points of view. Please feel free to share your point of view even if it differs from what others have said. We're on a first name basis, you don't need to agree with others, but you must listen respectfully as others share their views. We learn from you (positive and negative), not trying to achieve consensus but we’re gathering information. Thus keep in mind that we're just as interested in negative comments as positive comments, and at times the negative comments are the most helpful, we don't want to miss any of your comments. People often say very helpful things in these discussions and we can't write fast enough to get them all down, and we won't use any names in our reports. You may be assured of complete confidentiality. We ask that you turn off your phones, if you cannot and if you must respond to a call, please do so as quietly as possible and rejoin us as quickly as you can. My role as moderator will be to guide the discussion, you talk to each other, one person speaking at a time. You were invited because you're familiar with what HRM (strategy, practices) look like in hospital and health systems in Ethiopia, because of your expertise, or experiences that can help us.

I am distributing the Participant Information Sheet, the Consent Form, pseudo name cards with marker, and the Sign-In Sheet with a few quick demographic questions (age, gender, cadre, current role, type of hospital, yrs at hospital facility) around to the group while we are introducing each other .

**Our topic is HRM in Ethiopian Hospitals.** The results will be used for advancing the understanding on how HRM strategies evolve in the Ethiopian hospitals setting and for informing hospital managers and health policy makers. I am asking the group to suggest some ground rules. Let us make sure the following are on the list of ground rules: Everyone should participate; stay with the group and please don’t have side conversations, turn off cell phones if possible, have fun,…

I’ve got a number of questions that I want to ask and when needed to probe for specific examples or elaborations on the topics, but my role is to guide the discussion and really to listen and finally debrief you after the FGD. Thus I would like to moderate discussion on several topics that relate how HR strategy is developed in Ethiopia hospitals.

**Let’s start introducing ourselves and then start the discussion by talking about**

- - your job and responsibilities within your hospital/setting?
  - your educational background and experiences in this and similar organizations?
  - How long do you work in this hospital, in the current position/role?
  - What is your relationship to the HR strategy?

**Now, let us proceed to the five main research topics/themes and their sub-topics**

**1. Institutional Mechanisms [from health system, regulation & legal, political, cultural & societal contexts)**

- Which characteristics and developments in the health system impact the way you manage the hospital and especially your employees (professionals and staff)? Think for example of hiring, salary, working hours and conditions,…..
- Which governmental regulations and laws impact the way you manage the hospital and its workforce? (e.g. national labor laws and regulations, regulation for healthcare institutions)
- What political aspects are influential on your human resource management? Could be your direct relationships, or the relationships of your institution, e.g. with Health Bureau, University, Ministry, Politicians,…
- How do societal and cultural characteristics and developments impact your management? Think for example on (changing) demand and expectations, economic developments, poverty, health literacy,**…**

**2. Heritage Mechanisms (internal factors)**

- Can you sketch the organizational structure? Is there a dedicated HR department? Where is it positioned?
- What responsibilities are with the HR department? What HR responsibilities are with executive management? What with the line management?
- What is the history of this hospital, especially the recent history? What is special? What are the strengths, especially in workforce, skills, knowledge? What are challenges in HRM, workforce, skills, knowledge,….linkage of HR practices to outcomes
- How would you describe the organizational culture?

**3. Competitive Mechanisms :**

- - What services do you offer? What do you offer especially? What is it that you don’t offer?
  - Can you describe the market, i.e. your customers and your competitors?
  - What are the main developments and innovations in the market? Which technologies matter?
  - Which skills are important? Are you competing for skilled personal? Do they leave for the competitor?

**4. ‘Organizational capabilities’ and ‘Legitimacy’ related factors :**

- - Do you achieve the desired health outcomes for your patients (are you effective)?
  - Are you responsive? Can you instantly address urgent patients? Do you have short waiting times?
  - Are you efficient? Do you have low cost? OR are you rather focused on being high quality for instance?
- Do you learn new technology and procedures easily? Can you innovate-HRM practices?
- Do you manage to be compliant with all regulations on hospital care? Where are the challenges? How is related to human resource management?
- Do you manage to be compliant with all regulations for labor / employee relations? Where are the challenges?
- Can you get all work done within the skills and time of the corresponding employees?
- Can you divide work and manage employees equally and fairly?
- Are employees involved in decision making? Do you think they feel engaged? Involved?

**5. Leeway or key decision makers in employment relationship**

- - Who are the key decision makers in general hospital management?
  - Who decides on HRM (strategy)?
  - Could you tell me about your mandate/authority?
- Who decides on job description? Who on salary system and salary scale for health professionals? On salaries, promotions, allowances, incentives? Moonlighting? Hiring/firing?
- What promotes, what hinders the responsibilities of decision maker/s in this hospital?
- Which factors influence the choices and implementation of HR strategy?

**Thanks a lot for your time and cooperation**!

**Participant Information Sheet (PIS) and Consent Form (CF) for FGD participants**

***Dear madam/sir,***

Below, I wish to briefly introduce you *why this study is being done and what it will involve.*

To introduce myself and aim of the study, I am Philipos Petros conducting research on SHRM practices in Ethiopian hospitals. The aim of the study is to hear your experiences and opinion so as to advance our understanding on how HRM strategies evolve in the Ethiopian hospitals setting, how HR practices-outcomes related, how HRM -performance elements (effectiveness, efficiency, quality, waiting time, staff and patient satisfaction, ..) related. To this end, I and the research team member would like to conduct group discussion, and thank you in advance for your cooperation in sparing your busy schedule. I would like to ask you questions/professional experiences about several topics that relate how HR strategy is developed in your and/or other hospital. There are no good or wrong reflections/answers to the questions. This discussion will approximately take 90 minutes of your time. We are asking your permission to audiotape the group discussion, which do not aim to identify and describe your name, personality but only for research purpose. *Tapes will be identified only by a code, and will not be used or made available for any purposes other than the research project. These tapes will be destroyed at the end of the study. Y*our active participation is instrumental and your responses will be kept strictly confidential.

You have been chosen and approached because of your experiences and roles associated to this research and societal implications to hospitals and health system. We humbly let you know that taking part is entirely voluntary, beneficial to improve societal health, HRM in hospitals and organizational performance. There is no intended personal benefit (but social) in taking part, has no risks and that refusal or withdrawal will involve no penalty or loss, now or in the future but your information is vital. Thus, I kindly expect you will give genuine information, experiences and opinions. We would be happy to share a copy of (if published) results where you will not be identified in any report or publication. *The project has received ethical approval certificate from the Ethiopian Public Health Institute (EPHI) of the Federal Ministry of Health.*

*Contact Philipos Petros (Principal Investigator, 0913526545;* [multidisciplinary3@gmail.com](mailto:multidisciplinary3@gmail.com)*) for further information.*

**Consent Form**

- I have been debriefed, read and understood the Participant Information Sheet;
- I have been given the opportunity to ask questions and have had them answered to my satisfaction;
- I agree to take part in this research project;
- I understand that my participation is voluntary and I have been informed that I am free to withdraw at any time without giving a reason;
- I understand and agree to consent for EUR and scientific procedures for handling any personal data (e.g. confidentiality, anonymisation, etc.);
- I understand and agree to consent to proposals for data storage, archiving, sharing and re-use for future research;
- I understand and agree to consent to any planned audio or visual recording.

I kindly ask you to sign, print your name and date this form

| **Print your name** | **Signature** | **Date (GC)** |
| --- | --- | --- |
|  |  |  |
